# Activin A expression regulates multipotency of mesenchymal progenitor cells

**DOI:** 10.1186/scrt11

**Published:** 2010-05-04

**Authors:** Farida Djouad, Wesley M Jackson, Brent E Bobick, Sasa Janjanin, Yingjie Song, George TJ Huang, Rocky S Tuan

**Affiliations:** 1 Cartilage Biology and Orthopaedics Branch, National Institute of Arthritis and Musculoskeletal and Skin Diseases, National Institutes of Health, Department of Health and Human Services, 9000 Rockville Pike, Bethesda, Maryland 20892, USA; 2 Current address: Inserm U 844, Hôpital Saint-Eloi, Bâtiment INM, 34295 Montpellier, France; 3 Current address: Department of Biological Sciences, 2500 University Drive NW, Calgary, Alberta, T2N 1N4, Canada; 4 Current address: Department of Otorhinolaryngology, Head & Neck Surgery, Zagreb Clinical Hospital Centar, Zagreb University School of Medicine, Salata 4, 10000 Zagreb, Croatia; 5 Current address: Department of Molecular and Cell Biology, Boston University Henry M. Goldman School of Dental Medicine, 72 East Concord Street, Boston, MA 02118, USA; 6 Current address: Department of Orthopaedic Surgery, Center for Cellular and Molecular Engineering, University of Pittsburgh School of Medicine, 450 Technology Drive, Room 221, Pittsburgh, PA 15219, USA

## Abstract

**Introduction:**

Bone marrow (BM) stroma currently represents the most common and investigated source of mesenchymal progenitor cells (MPCs); however, comparable adult progenitor or stem cells have also been isolated from a wide variety of tissues. This study aims to assess the functional similarities of MPCs from different tissues and to identify specific factor(s) related to their multipotency.

**Methods:**

For this purpose, we directly compared MPCs isolated from different adult tissues, including bone marrow, tonsil, muscle, and dental pulp. We first examined and compared proliferation rates, immunomodulatory properties, and multidifferentiation potential of these MPCs *in vitro*. Next, we specifically evaluated activin A expression profile and activin A:follistatin ratio in MPCs from the four sources.

**Results:**

The multidifferentiation potential of the MPCs is correlated with activin A level and/or the activin A:follistatin ratio. Interestingly, by siRNA-mediated activin A knockdown, activin A was shown to be required for the chondrogenic and osteogenic differentiation of MPCs. These findings strongly suggest that activin A has a pivotal differentiation-related role in the early stages of chondrogenesis and osteogenesis while inhibiting adipogenesis of MPCs.

**Conclusions:**

This comparative analysis of MPCs from different tissue sources also identifies bone marrow-derived MPCs as the most potent MPCs in terms of multilineage differentiation and immunosuppression, two key requirements in cell-based regenerative medicine. In addition, this study implicates the significance of activin A as a functional marker of MPC identity.

## Introduction

Mesenchymal progenitor cells (MPCs) are multipotent cells, derived from various adult tissues, that are capable of differentiating into several mesenchymal lineages, including osteoblasts, chondroblasts, and adipocytes. A large body of data suggested MPCs as a promising candidate cell type applicable for repair and regeneration of a variety of mesenchymal tissues such as bone, cartilage, and muscle [1,2]. MPCs were initially identified and isolated from bone marrow (BM) and are characterized by the expression of a number of cell surface markers [3-5]. Based on their clonogenic and multipotent differentiation activities, to date, MPCs have been isolated from a number of adult tissues, including trabecular bone [6], fat [7,8], synovium [9,10], skin [11], thymus [11,12], periodontal ligament [13], as well as prenatal and perinatal sources such as umbilical cord blood [14], umbilical cord [15], palatine tonsil [16], and placenta [17]. The diversity of sources facilitates MPC accessibility, but also raises questions about possible phenotypic and functional discrepancies that must be addressed for their clinical use.

The transforming growth factor-β (TGF-β) superfamily of secreted factors includes TGF-β, activins, Nodal, and bone morphogenetic proteins (BMPs). The activation of the TGF-β/activin/Nodal signaling pathway through SMAD2/3 is associated with the pluripotency of human embryonic stem cells (hESCs) and is required for the maintenance of their undifferentiated state [18]. Through the induction of Oct4, Nanog, Nodal, Wnt3, basic fibroblast growth factor (FGF-2), and FGF-8, Activin A was shown to be a key regulator for the "stemness" maintenance of hESCs [19]. Activin A, like other members of the TGF-β superfamily, has also been described to affect embryogenesis, hematopoiesis, and angiogenesis [20-22]. The actions of activin A are determined by a balance of the levels of activin A and its inhibitor, follistatin (FS). FS is a natural antagonist that binds activin with high affinity and neutralizes its biologic activities by preventing activin interaction with its membrane receptors [23,24]. Activin ligands exist in three forms: homodimers of the βA and βB protein subunits constitute activin A and activin B, respectively, and a heterodimer of βA and βB protein subunits represents activin AB. These ligands signal by binding to specific serine/threonine kinase type II (ActRIIA and ActRIIB) receptors. In the adult, activin βA subunit mRNA is produced in BM [25] and, like TGF-β [26] and BMPs, activin A is abundantly localized in bone matrix [27,28]. BM-derived stromal fibroblasts were reported to be the major source of activin A and FS in the BM [29]. The role of activin A in bone metabolism has been evaluated in several studies. Although an inhibitory effect of activin A on osteoblastic differentiation in rat and murine osteoblasts was described [30,31], activin A was also shown to stimulate osteoblastogenesis in murine bone marrow cultures and, *in vivo*, promotes bone formation and fracture healing in rodents [27-29,32]. Interestingly, it was recently shown that the osteoblastic differentiation of MPCs induced by BMP-2 involved an activin-dominant microenvironment, whereas adipogenic differentiation of MPCs in the presence of dexamethasone occurred in an FS-dominant microenvironment [33]. In this study, the authors suggested that activin A inhibits adipogenesis by affecting the adipocyte transcriptional factor in favor of osteoblastic differentiation [33,34]. Activin βA also was detected in developing cartilage and identified as another member of the TGF-β superfamily involved in the induction of limb chondrogenesis [35,36]. Therefore, activin A is likely to have pleiotropic functions, including an essential role in sustaining hESC pluripotency, and to play a critical role during skeletogenesis.

In addition to the MPCs derived from BM, MPCs that show multidifferentiation potential have also been isolated from various tissues. However, compared with BM-MPCs, these MPCs of different tissue origins may have different cellular properties and therefore present differential clinical applicability for regenerative medicine. It is, therefore, desirable to identify a factor that differentially correlates with and provides a functional characterization of the individual "stem cell" state of the MPCs from various sources. In this regard, activin A, which has been shown to play a role in regulating MPC properties, may be considered as a candidate factor and should be examined for its expression and functional correlation with the activities of various MPCs. For this purpose, we have undertaken a comparative study of MPCs derived from four different tissue sources, including BM, palatine tonsil (T), dental pulp (DP), and skeletal muscle (M). In line with the current state of knowledge of the biology of BM-derived MPCs, we carried out direct comparisons of the phenotypic and functional properties of MPCs isolated from these different sources. Specifically, we tested the hypothesis that activin A may function as a critical regulatory factor for MPCs multipotency. We further dissected the requirement of activin A in human MPCs induction toward differentiation into chondrocytes, osteoblasts, and adipocytes. Our findings showed that activin A expression level in MPCs discriminates MPCs from different sources in terms of proliferation capacity and differentiation potential. In addition to a possible role in MPC identity, activin A also appears to play a pivotal role in the early stages of MPC chondrogenesis and osteogenesis while inhibiting adipogenesis. Furthermore, our results show that among the four tissue sources compared, BM-derived MPCs have the highest differentiation potency and represent the most desirable cell type for regenerative medicine.

## Materials and methods

### MPC isolation and culture

#### Tonsil-derived MPCs (T-MPCs)

With Institutional Review Board (IRB) approval (George Washington University, Washington, DC), tonsils were obtained after informed consent from patients undergoing tonsillectomy as a result of recurrent episodes of acute tonsillitis. The tissue was minced, digested, and processed by direct plating, as described previously [16]. Expansion medium consisting of Dulbecco's modified Eagle's medium (DMEM) (Invitrogen Corporation, Carlsbad, CA, USA) with 10% fetal bovine serum (FBS) from selected lots (HyClone) and antibiotics (50 μg/ml streptomycin and 50 IU/ml penicillin; Invitrogen).

#### Bone marrow-derived MPCs (BM-MPCs)

Bone marrow (BM) was obtained after informed consent from patients (39 to 58 years old) undergoing lower extremity reconstructive surgery with IRB approval (University of Washington, Seattle, WA, and George Washington University) and was processed by direct plating, as described previously [37]. BM aspirates were plated overnight in T-150 cm^2 ^culture flasks in the same expansion medium as T-MPCs, and adherent cells were obtained similarly. Both T-MPCs and BM-MPCs were culture-expanded in expansion medium at 37°C and 5% CO_2 _by using T-150 Triple Flasks (Nunc), and medium changes were done twice weekly.

#### Traumatized muscle-derived MPCs (M-MPCs)

Traumatically injured muscle was collected with IRB approval from the Walter Reed Army Medical Center (WRAMC, Washington, DC). Patients considered for inclusion in this study had been exposed to orthopedic trauma and sustained extensive soft tissue extremity wounds, and informed consent was obtained from each patient before tissue collection. These patients arrived at WRAMC approximately 3-7 days after injury, and tissue was collected during debridement and irrigation procedures. The tissue was dissected, minced, digested, and plated as described previously [38,39]. The adherent cells were cultured in the same expansion medium as T-MPCs. For the first 3 days, the cells were washed with Hank's Balanced Salt Solution (HBSS), and the medium was replaced. The cells were subcultured into new flasks after tightly packed colony-forming units (CFUs) were observed, and the cells were cultured in basal expansion or Growth Medium (GM: DMEM supplemented with 10% FBS and 1 unit/ml of antibiotics) from that point forward. Subsequent subcultures were performed when the cells were approximately 85% confluent.

#### Dental pulp-derived MPCs (DP-MPCs)

Tooth sample collection and the growth of pulp cells followed protocols described previously [40,41]. Extraction of third molars from healthy patients (aged 16-26 years) in the Department of Oral Surgery conformed to a protocol (H-29892) approved by the University of Maryland Medical School IRB. Pulp-cell cultures were established through enzyme digestion. Minced pulp tissues were digested in a solution of 3 mg/ml collagenase type I and 4 mg/ml dispase (Sigma) for 30-60 min at 37°C. Cell suspensions were obtained by passing the digested tissues through a 70-μm cell strainer (Becton-Dickinson). Single-cell suspensions (1 × 10^5 ^cells/flask) were seeded in 5 × 10-cm culture flasks containing Eagle's minimum essential medium alpha modification (α-MEM) supplemented with 20% FBS, 2 mmol/L L-glutamine, 100 μmol/L L-ascorbic acid-2-phosphate, 100 U/ml penicillin-G, 100 μg/ml streptomycin, and 0.25 μg/ml fungizone (Gemini Bio-Products) and maintained under 5% CO_2 _at 37°C. Clonogenic cells were grown to 70% confluence, passaged at a 1:3 ratio, and cultured in the same expansion medium as T-MPCs.

### Cell-proliferation assay

Cell-proliferation assays were performed by using the Cell Counting Kit-8 (Dojindo, Kumamoto, Japan). Cells were plated in 96-well plates at 1 × 10^4 ^cells per well and cultured in the basal growth medium. At the indicated time points, cell numbers in triplicate wells were measured spectrophotometrically as A_450 _values of reduced WST-8 (2-(2-methoxy-4-nitrophenyl)-3-(4-nitrophenyl)-5-(2,4 disulfophenyl)-2H-tetrazolium, monosodium salt).

### Flow cytometry

For flow cytometry, BM-MPCs, T-MPCs, DP-MPCs, and M-MPCs (>2 × 10^5 ^cells) were washed and resuspended in phosphate-buffered saline (PBS) + 0.1% FBS (PF), containing saturating concentrations (1:100 dilution) of the following conjugated mouse IgG_1,κ _anti-human monoclonal antibodies (BD Biosciences): CD14-PE, CD34-PE, CD45-FITC, CD73-PE, CD90-FITC, CD105-PE for 1 h at 4°C. Cell suspensions were washed twice and resuspended in PF for analysis on a flow cytometer (FACSCalibur, BD Biosciences) by using the CellQuest ProTM software (BD Biosciences).

### *In vitro *differentiation

MPCs were induced to undergo adipogenic, osteogenic, and chondrogenic differentiation, as described previously [37]. For adipogenic differentiation, cells were seeded into six-well tissue-culture plates at a density of 20,000 cells/cm^2 ^and treated for 3 weeks with adipogenic medium, consisting of DMEM with 10% FBS, and supplemented with 0.5 mmol/L 3-isobutyl-1-methylxanthine (IBMX), 1 μg/ml insulin, and 1 μmol/L dexamethasone (all from Sigma). For osteogenic differentiation, cells were seeded into six-well plates (Corning) at a density of 20,000 cells/cm^2^, and treated for 3 weeks with osteogenic medium, consisting of DMEM with 10% FBS, and supplemented with 10 mmol/L β-glycerolphosphate, 10 nmol/L dexamethasone, 50 μg/ml ascorbic acid-2-phosphate, and 10 nmol/L 1,25 dihydroxyvitamin D_3 _(Biomol International L.P., Plymouth Meeting, PA). To induce chondrogenic differentiation, 96-microwell polypropylene plates (Nunc, Roskilde, Denmark) were seeded with 300,000 cells per well, and cell pellets formed by centrifugation at 1,100 rpm for 6 minutes. The pellet cultures were treated for 3 weeks with chondrogenic medium, consisting of high-glucose DMEM supplemented with 100 nmol/L dexamethasone, 50 μg/ml ascorbic acid-2-phosphate, 100 μg/ml sodium pyruvate, 40 μg/ml L-proline, 10 ng/ml recombinant human transforming growth factor-β3 (TGF-β3; R&D Systems, Minneapolis, MN), and 50 mg/ml ITS-premix stock (BD Biosciences).

### Histochemistry

#### Oil Red O Staining

Three-week adipogenic cultures of MPCs were rinsed twice with PBS, fixed in 4% buffered paraformaldehyde, stained with Oil red O (Sigma) for 5 minutes at room temperature, and counterstained with Harris-Hematoxylin Solution (Sigma), to visualize lipid droplets.

#### Alkaline phosphatase and alizarin red S staining

MPCs cultured for 3 weeks in osteogenic medium were fixed in 4% paraformaldehyde, rinsed, and stained for alkaline phosphatase with Naphthol AS-BI substrate (Sigma) for 30 minutes at 37°C in the dark. Osteogenic cultures were also fixed with 60% isopropyl alcohol and stained for 3 minutes with 2% (wt/vol) Alizarin Red S (Rowley) at pH 4.2 to detect mineralization.

#### Alcian blue and picrosirius red staining

Chondrogenic cell pellets were fixed in 4% buffered paraformaldehyde, rinsed with PBS, serially dehydrated, paraffin embedded, and sectioned at 10-μm thickness. Sections were stained with Alcian blue (pH 1.0) to detect sulfated proteoglycans and with Picrosirius Red (0.1% wt/vol in saturated picric acid) to visualize collagen deposition.

### Lymphocyte proliferative assay

Peripheral blood from healthy human donors was collected into heparinized containers (BD Biosciences), and peripheral blood mononuclear cells (PBMCs) were isolated by Ficoll-Hypaque density gradient centrifugation. Responder human PBMCs were resuspended in RPMI 1640 medium containing 10% FBS, 2 mmol/L glutamine, 100 U/ml penicillin, and 100 μg/ml streptomycin, 0.1 mmol/L nonessential amino acids, 1 mmol/L sodium pyruvate, 20 mmol/L HEPES, and 50 μmol/L 2-mercaptoethanol (Invitrogen). PBMCs were seeded in triplicates at 1 × 10^5 ^cells/100 μl per well in 96-well round-bottom plates (BD Biosciences). PHA was used at 5 μg/ml as a positive control mitogen to induce T-cell proliferation. MPCs (5 × 10^4^cells) were added to obtain a final volume of 300 μl. After 3 days of incubation, 1 μCi/well [^3^H]-thymidine (GE Healthcare) was added, and radioactivity incorporation over a 12-hour period was determined by liquid scintillation counting. All experiments were performed in triplicate and repeated at least twice.

### Immunohistochemistry

Chondrogenic cell pellets were fixed in 4% buffered paraformaldehyde, rinsed with PBS, serially dehydrated, paraffin embedded, and sectioned at 10-μm thickness. Sections were digested with 1 mg/ml hyaluronidase (Sigma) or 20 μg/ml proteinase K (Roche Diagnostics) in 10 mmol/L Tris-HCl (pH 7.5) for 30 minutes at 37°C. The sections were then incubated overnight at room temperature with the following antibodies in Tris-buffered saline containing 0.1% bovine serum albumin: antibodies to aggrecan and collagen type II (Developmental Studies Hybridoma Bank), and antibodies to fibronectin (R&D). Immunostaining was detected by using the chromogenic streptavidin-peroxidase Histostain SP Kit for DAB (Zymed Laboratories, San Francisco, CA), and imaged by using an inverted microscope equipped with a color charge-coupled device camera.

### RNA isolation and real-time reverse transcription-polymerase chain reaction (RT-PCR)

RNA was extracted from monolayer cultures of BM-MPCs, T-MPCs, DP-MPCs and, M-MPCs by using the RNeasy Kit (Qiagen). Total RNA (1 μg) was reverse transcribed by using the Multiscribe reverse transcriptase (Applied Biosystems). Real-time PCR reactions were performed as previously described [42]. Gene-specific primers (forward and reverse) were designed based on GenBank cDNA sequences (primer sequences available on request). Specific transcript levels were normalized by comparison with the housekeeping gene, glyceraldehyde-3-phosphate dehydrogenase (GAPDH).

### Western blot

MPCs cultures were treated with activin A (10 ng/ml) for 48 hours, and homogenized in RIPA lysis buffer (Sigma) supplemented with protease and phosphatase inhibitors. Extracted proteins were separated on 12% polyacrylamide gels and electroblotted. The blots were immunoprobed for GAPDH and Sox-2 (Santa Cruz, CA). Appropriate horseradish peroxidase-conjugated secondary antibodies were used for detection.

### Activin A quantification

Activin A in culture supernatants was quantified by using a commercially available enzyme-linked immunosorbent assay kit (R&D Systems) according to the manufacturer's protocol.

### siRNA transfection

Small interfering RNA (siRNA) duplexes designed against human *inhibin, beta A *(activin A, activin AB alpha polypeptide), and the negative control siRNA obtained from Ambion (Applied Biosystems/Ambion, Austin, TX) were reconstituted by following the manufacturer's guidelines. siRNAs were transfected into BM-MPCs by electroporation with a total of 750,000 cells in 300 μl of siPORT electroporation buffer containing 45 μl of 22.5 μg siRNA by using the BTX ECM 830 Electro Square Porator System (Harvard Apparatus). In brief, the mixture was transferred to a 2-mm gap BTX electroporation cuvette, which was then placed in the apparatus and exposed to two identical square-wave pulses (parameters: 1,075 V, 105-μs pulse length, 5-second duration between pulses). After electroporation, the cuvettes were incubated for 15 minutes at 37°C in a 5% CO_2_/95% air atmosphere. The transfected MPCs were then maintained for 24 hours in culture medium under 5% CO_2 _at 37°C before differentiation analyses.

### Statistics

Data from the proliferation, RT-PCR, and ELISA experiments were analyzed statistically by using Student's *t *test, with statistical significance set at *P *< 0.05.

## Results

### Differential cell properties among mesenchymal progenitor cells from various tissue sources

Before examining the role of activin A, the four types of MPCs (BM, T, DP, and M) were first examined in terms of several key properties.

#### Cell surface-marker expression

To confirm that these MPCs express typical cell-surface markers, we performed fluorescence activated cell sorting (FACS) analysis. It was revealed that all MPCs were negative for CD14, CD34, and CD45 expression and positive for CD73, CD90, and CD105 (Table 1).

**Table 1:** Surface epitope phenotype of bone marrow-, tonsil-, muscle-, and dental pulp-derived mesenchymal progenitor cells*

	BM-MPCs	T-MPCs	M-MPCs	DP-MPCs
CD14	-	-	-	-
CD34	-	-	-	-
CD45	-	-	-	-
CD73	+	+	+	+
CD90	+	+	+	+
CD105	+	+	+	+

*Surface epitopes or CD markers identified on the basis of immuoreactivity analyzed by fluorescence-activated cell sorting (FACS). FACS analysis showed that more than 95% of these cells were negative (-) for the expression of CD14, CD34, and CD45, and were positive (+) for CD73, CD90 and, CD105. Controls consisted of fluorescence observed with isotypic controls. BM, bone marrow; DP, dental pulp; M, muscle; MPC, mesenchymal progenitor cell; T, tonsil.

#### T-MPCs have a higher proliferation rate than other MPCs

**Figure 1:**
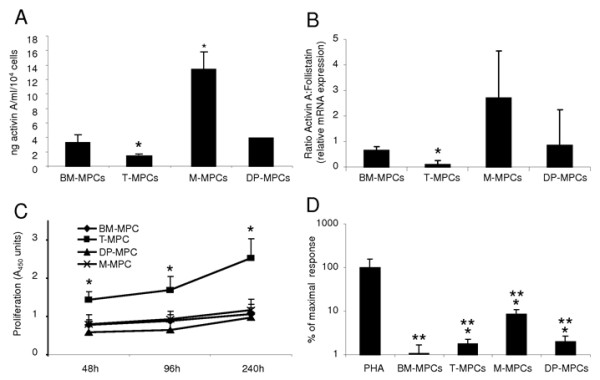
**Activin A expression profile, proliferative rate, and immunosuppressive characteristics of MPCs from different tissue sources**. **(a) **ELISA quantification of secreted activin A. Results are expressed as the mean of secretion in 48-hour supernatants of three cell cultures (mean ± S.D.). **(b) **Activin A:follistatin ratio by real-time RT-PCR. **(c) **Cell-proliferation rate determined by using CCK-8. BM-MPCs, T-MPCs, M-MPCs, and DP-MPCs were plated in 96-well plates at 1 × 10^4 ^cells per well and cultured in growth medium for 48, 96, 144, and 240 hours. **(d) **Proliferative activity of T cells stimulated with PHA. Responding PBMCs were stimulated with PHA at 5 μg/ml in the presence or absence of 5 × 10^4 ^MPCs. The proliferative response of T cells is represented as a percentage of PHA control (mean ± SD of triplicates). *P ≤ 0.05, versus BM-MPCs; **P ≤ 0.05 versus PHA.

Analysis of cell density as a function of culture time (48, 96, 144, or 240 hours) in growth medium revealed that among the four different MPC populations tested, T-MPCs proliferated at a significantly higher rate than did MPCs derived from the other tissues (Figure 1c).

#### BM-MPCs display the strongest chondrogenic and adipogenic potency, whereas DP-MCPs exhibit the highest level of matrix mineralization

**Figure 2:**
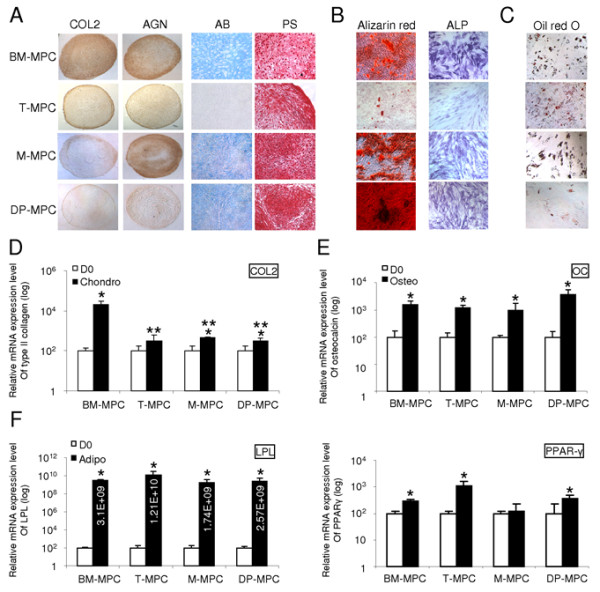
***In vitro*-induced differentiation of MPCs from different tissue sources**. **(a-d) **Chondrogenic differentiation evaluated after 21 days in micropellet culture. **(a) **Immunohistochemical analysis of cartilage markers (COL2 and AGN) and alcian blue (AB) and picrosirius (PS) staining in pellet cultures of MPCs. **(d) **Expression of COL2, a specific marker for chondrogenesis, measured in differentiated MPC cultures compared with day 0 (D0) cultures, determined by quantitative RT-PCR with GAPDH as an internal control. **(b-e) **Osteogenic differentiation of MPCs assessed after 21-day culture in osteogenic medium. (b) All MPCs tested exhibited ALP activity and produced a mineralized matrix stained with alizarin red. T-MPCs displayed less alizarin red staining compared with BM-MPCs, M-MPCs, and DP-MPCs. **(e) **Significant upregulation of the expression of osteocalcin (OC), an osteoblast-specific gene, compared with undifferentiated MPCs was observed after osteogenic induction of BM-MPCs, T-MPCs, M-MPCs, and DP-MPCs compared with D0 cultures. **(c-f) **Adipogenic differentiation of MPCs cultured in adipogenic medium for 21 days. **(c) **All MPCs tested exhibited lipid-containing intracellular vacuoles that stained with oil red O at the end of the differentiation period. **(f) **MPCs cultured in adipogenic medium upregulated the expression of adipocyte-specific genes (PPAR-γ and LPL) compared with MPCs at D0. Quantitative RT-PCR data represent the mean ± SD in three independent experiments. *P ≤ 0.05, versus D0; **P ≤ 0.05, versus BM-MPCs.

We compared the differentiation potential of MPCs derived from bone marrow, tonsil, muscle, and dental pulp with the chondrogenic, osteogenic, and adipogenic lineages. Cultures of MPCs derived from the four tissues under chondrogenic conditions produced pellets with a similar morphology and a shiny surface that is characteristic of cartilaginous pellets. At day 21 of chondrogenesis, alcian blue (AB) and picrosirius histologic staining of the chondrogenic pellets derived from BM-MPCs, M-MPCs, and DP-MPCs all revealed the presence of a sulfated proteoglycan- and collagen-rich extracellular matrix. In comparison, in T-MPC pellets, the matrix contained considerably less sulfated proteoglycans, whereas collagen staining was highly positive (Figure 2a). Immunohistochemistry showed that all MPC pellets produced collagen type II (COL2) and aggrecan (AGN), although the staining intensities for both COL2 and AGN were higher in BM-MPCs compared with those of T-MPCs, M-MPCs, and DP-MPCs (Figure 2a). Quantitative RT-PCR analysis of day 21 cultures showed that, in response to the chondrogenic culture conditions and induction medium, expression of COL2 was upregulated in all cultures, with the highest level in BM-MPCs (Figure 2d).

Although to a lesser extent for T-MPCs, MPCs from the four tissue sources cultured for 21 days in osteogenic medium exhibited increased ALP activity and produced a mineralized matrix that stained positive with alizarin red, with the highest level of mineralization seen in DP-MPCs cultures (Figure 2b). Under the same osteogenic conditions, gene expression of osteocalcin (OC), associated with mature osteoblasts, was significantly increased compared with the undifferentiated cells at day zero (D0) (Figure 2e).

MPCs cultured in adipogenic medium formed characteristic intracellular lipid-rich vacuoles that stained positive with oil red O. Compared with BM-MPCs, T-MPCs, and M-MPCs, DP-MPCs displayed a lesser ability to form lipid vacuoles (Figure 2c). MPCs derived from the different tissue sources under adipogenic induction also upregulated the expression of peroxisome proliferator-activated receptor-γ (PPAR-γ), a master regulator of adipogenesis, as well as lipoprotein lipase (LPL) (Figure 2f). To summarize, the nonequivalence in differentiation potency of the MPCs included (a) BM-MPCs displayed a significantly greater chondrogenic potential; (b) DP-MPCs presented a higher capacity for extracellular matrix mineralization; and (c) T-MPCs showed an inferior ability to differentiate into chondrocytes and osteoblasts but a high adipogenic potential.

#### BM-MPCs exhibit greater immunosuppressive properties

In addition to their multilineage differentiation potential, MPCs have been shown to display immunoregulatory properties that have prompted consideration of their use in bone marrow transplantation [3,43,44]. Although the exact immunosuppressive mechanisms of MPCs are unknown, the capacity of MPCs to suppress T-cell proliferation stimulated by allogeneic lymphocytes, dendritic cells, and phytohemagglutinin (PHA) is well documented [45,46]. The immunosuppressive properties of the MPCs derived from different tissue sources were tested in a lymphocyte proliferative assay by using human peripheral blood mononuclear cells (PBMCs) from healthy donors and stimulated with PHA, a mitogen. The addition of BM-MPCs, T-MPCs, M-MPCs, and DP-MPCs to the activated PBMCs inhibited the PHA-induced proliferative response of PBMCs (Figure 1d). The immunomodulatory activity of BM-MPCs was significantly more pronounced than that of T-MPCs, M-MPCs, or DP-MPCs (Figure 1d).

### Involvement of activin A in the regulation of mesenchymal progenitor cell properties

Given that the MPCs from the various tissue sources exhibit differences in terms of cell proliferation, multipotency, and immunomodulatory properties, we next examined the involvement of activin A by analyzing whether activin A (a) is expressed differentially in these MPC populations, (b) regulates MPC differentiation potential, and (c) regulates Sox2 expression in the MPCs.

#### Activin A distinguishes mesenchymal progenitor cells from different tissue sources

ELISA was used to quantify activin A secretion in the supernatants of MPCs derived from the four tissue sources. M-MPCs and BM-MPCs expressed the highest levels of activin A, whereas T-MPCs had the lowest level (Figure 1a). Next, the activin A:FS ratio was determined, because activin A bioactivity is also regulated by FS. Quantitative RT-PCR results showed that the activin A:FS level was also significantly lower in T-MPCs than in the other MPCs tested (Figure 1b).

#### Activin A expression levels regulate mesenchymal progenitor-cell differentiation potential

**Figure 3:**
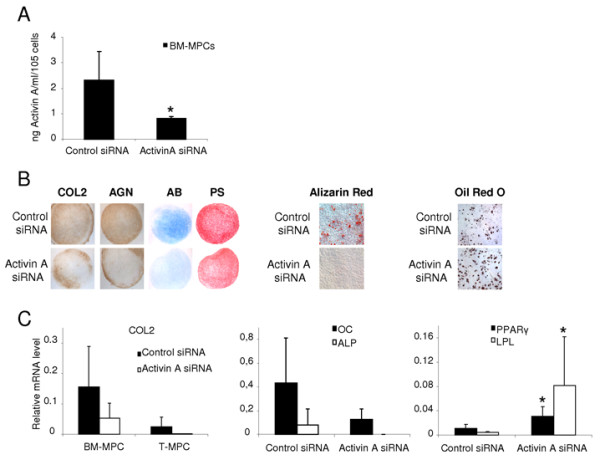
**Role of activin A in MPC multipotency**. BM-MPCs transiently transfected either with control siRNA or with activin A siRNA were analyzed. **(a) **Activin A secretion level in the supernatant of transfected BM-MPCs quantified by ELISA 3 days (D3) after transfection showed the efficiency of knockdown by using activin A siRNA. **(b, c) **Effect of activin A knockdown on BM-MPC and T-MPC chondrogenic potential. Chondrogenic differentiation was evaluated after 21 days in micropellet culture. **(b) **Immunohistochemical analysis of cartilage markers (COL2 and AGN) in pellet cultures of BM-MPCs and T-MPCs as well as alcian blue (AB) and picrosirius red (PS) staining revealed that, compared with the untransfected and Control siRNA, MPCs transfected with activin A siRNA exhibited less chondrogenic potential. **(c) **Quantitative RT-PCR also showed decreased expression of COL2 in MPCs transfected with activin A siRNA, compared with the cells transfected with control siRNA (c). **(b, c) **Effect of activin A knockdown on BM-MPC osteogenic potential. Both the expression levels of the osteogenic markers (OC and ALP), determined by quantitative RT-PCR, and alizarin red staining, revealed a decrease in the osteogenic activity of BM-MPCs with activin A knockdown. **(b, c) **Effect of activin A knockdown on BM-MPC adipogenic potential. Both expression levels of the adipogenic markers (PPAR-γ and LPL), determined by quantitative RT-PCR and oil red O staining, revealed an increase in the adipogenic potential of BM-MPCs with activin A knockdown, compared with BM-MPCs transfected with the irrelevant Control siRNA. RT-PCR data represent the mean ± SD of three independent experiments. *P ≤ 0.05, versus Control siRNA.

Because BM-MPCs exhibited high activin A expression level as well as the highest chondrogenic potential among the MPCs tested, we next determined the role of activin A in their multipotency by silencing of activin A in BM-MPCs. With the transfection protocol using 22.5 μg of Cy3-conjugated control siRNA, a transfection efficiency of 91.66 ± 1.36% at day 2 and of 57.36 ± 3.27% at day 7 was achieved. BM-MPCs were transfected with either activin A or control siRNA oligonucleotides. Analysis of culture supernatants demonstrated that activin A siRNA transfection significantly reduced activin A level (0.81 ± 0.08 ng/ml) compared with the si Control (2.32 ± 1.12 ng/ml) (Figure 3a). Twenty-four hours after transfection with gene-specific designed siRNA against activin A or with the Control siRNA, BM-MPCs were induced to differentiate toward the chondrogenic, osteoblastic and adipocytic lineages. On activin A knockdown, BM-MPCs displayed decreased chondrogenic and osteogenic potentials. By day 21, BM-MPCs transduced with activin A siRNA, and induced to differentiate into chondrocytes, formed a pellet similar to the untransfected cells but with a reduced production of COL2 and AGN. Histologic staining showed a clear decrease in sulfated proteoglycan content and in fibrous collagen matrix, as assessed by alcian blue and Picrosirius red staining, respectively (Figure 3b). This observation was supported by the decrease in COL2 mRNA expression in pellets formed with activin A siRNA transfected cells, as assessed by quantitative RT-PCR (Figure 3c). BM-MPCs lacking activin A and induced to undergo osteogenesis also displayed decreased mRNA-expression levels of osteoblastic markers such as OC and ALP (Figure 3c).

Activin A knockdown also affected matrix mineralization in osteogenically induced BM-MPCs compared with the untransfected cells (Figure 3b). Additionally, transfection of BM-MPCs with activin A siRNA enhanced their differentiation toward the adipocytic lineage. Compared with BM-MPCs transfected with the Control siRNA, activin A knockdown in BM-MPCs significantly increased the expression level of PPAR-γ and LPL (0.01 ± 0.007 to 0.03 ± 0.01 and 0.004 ± 0.001 to 0.08 ± 0.08, respectively) (Figure 3c). In summary, the transient knockdown of activin A in BM-MPCs at the early stages of the differentiation process appeared to stimulate adipogenesis while inhibiting chondrogenesis and osteogenesis.

#### Activin A increases Sox2 expression in BM-mesenchymal progenitor cells

**Figure 4:**
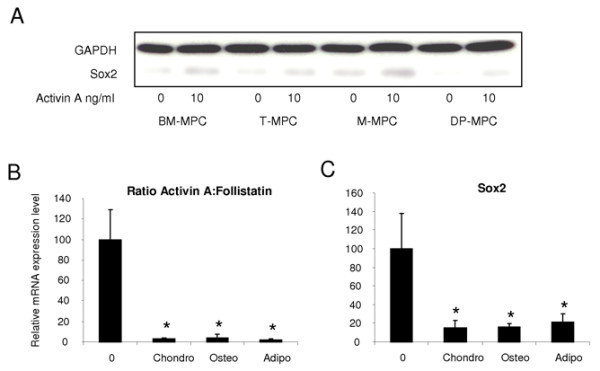
**Relation between Sox2 expression levels and activin A:follistatin expression ratio in MPCs from different tissue sources**. **(a) **Western blot analysis of Sox2 expression in BM-MPCs, T-MPCs, M-MPCs, and DP-MPCs before and after a 48-hour treatment with 10 ng/ml of activin A. **(b) **Activin A:follistatin expression ratio determined by quantitative RT-PCR in BM-MPCs before (day 0 (0)) and after their differentiation along chondrogenic, osteogenic, and adipogenic lineages. **(c) **mRNA expression levels of Sox2 in undifferentiated (day 0 (0)) and chondro-, osteo- and adipo-differentiated BM-MPCs. The values at day 0 (0) corresponding to the mRNA ratio of activin A:follistatin **(b) **to the mRNA level of Sox2 **(c) **relative to GAPDH were assigned the values of 100% ± SD. *P ≤ 0.05, versus Day 0.

Sox2, a transcription factor highly expressed in undifferentiated ES cells, has been shown to stabilize ES cells in a pluripotent state [47,48]. With Western blot analysis, we observed that although untreated MPCs expressed undetectable or low levels of Sox2 protein, its level increased 48 hours after the treatment with 10 ng/ml of activin A (Figure 4a).

The activin A:follistatin ratio was assessed by using quantitative RT-PCR before and after the differentiation of BM-MPCs into chondrocytes, osteoblasts, and adipocytes. The activin A:follistatin ratio was significantly lower in all differentiated cells compared with undifferentiated BM-MPCs (Figure 4b). In a similar manner, the Sox2 mRNA expression level was also significantly decreased in the differentiated cells compared with undifferentiated BM-MPCs (Figure 4c).

## Discussion

To realize the potential of MPCs in clinical applications, it is important to understand the biologic regulation of their multipotency. We attempted to gain insights into this by analyzing comparable MPCs derived from various tissue sources that have similar but non-identical characteristics and biologic activities, and we observed a requirement for activin A expression in the maintenance of MPC identity, as well as the regulation of MPC functions. Moreover, our findings strongly suggest the high potency of bone marrow-derived MPCs compared with MPCs from other tissue sources.

We showed that MPCs isolated from bone marrow, tonsil, muscle, and dental pulp all display phenotypic and functional properties that currently characterize MPCs. However, significant differences in activin A expression levels and, more important, in the activin A:follistatin ratios, which corroborate with MPC multipotency and immunosuppressive properties, were observed. Activins bind to the activin type IIA or type IIB receptors, leading to recruitment and phosphorylation of the activin type IB receptor, which in turn phosphorylates intracellular signaling proteins, Smad2 or Smad3. Activin A signaling is inhibited by extracellular action of follistatin, a soluble protein that functions as an activin-binding protein to prevent activin from interacting with its receptor. Activin A but not the activin A:follistatin ratio was significantly higher in M-MPCs compared with the other MPCs tested. In T-MPCs, both activin A level and activin A:follistatin ratio were significantly lower compared with the other MPCs. In parallel, we showed that the proliferative rate of T-MPCs was significantly higher compared with the other MPCs studied. This observation suggests that the endogenous high level of activin A might reduce the proliferation of BM-MPCs, M-MPCs, and DP-MPCs compared with that of T-MPCs. Although activin A is widely expressed by various cells, the effects of activin A are diverse, and cell type dependent. In general, activin A exerts antiproliferative effects on many cell types, including epithelial cells and vascular endothelial cells and lymphocytes [49,50]. However, several cell types, including fibroblast-like synoviocytes (FLS) and lung fibroblasts, proliferate in response to activin A [51,52]. In the study using FLS, the authors emphasized the differences between the amount of activin A produced by the FLS (<1 ng/ml) and the amount of exogenous activin A necessary to promote FLS proliferation (250 ng/ml).

Besides cell growth, activin A has been widely studied for its role in cell differentiation. Several studies demonstrated a role for activins in bone metabolism. During differentiation and bone formation, osteoblasts produce a characteristic extracellular matrix that eventually undergoes mineralization. Although activin A has been described to promote osteoblastogenesis in murine bone marrow cultures and to enhance *in vivo *bone formation and fracture healing in rodents [27-29,32], other reports described an inhibitory effect of activin on osteoblast differentiation in rat and murine osteoblasts [30,31]. These opposite effects of activin A in murine rodent models might be explained by the cell type used (that is, activin A is a potent regulator of osteogenesis with a positive effect on uncommitted progenitor cells and an inhibitory action on mature osteoblasts). This postulate is supported by the finding that activin A signaling in osteoblasts is tightly controlled in a differentiation-dependent manner [53]. Mineralizing osteoblast cultures showed decreased activin A production. Activin A signaling controls human osteoblast differentiation and function and inhibits matrix mineralization through mechanisms that include altered extracellular matrix composition and maturation. The expression of activin A in bone, taken together with its effect on mineralization *in vitro*, strongly suggests a biologically important role for activin A in human osteoblast differentiation and bone metabolism [53]. In this work, we observed that T-MPCs, with lower level of activin A expression compared with the other MPCs, also exhibit lower osteogenic potential, as shown by ALP activity and alizarin red staining. The involvement of activin A in the early events of osteogenesis is also supported by the effects of siRNA-mediated transient knockdown of activin A gene expression in BM-MPCs induced to undergo osteogenesis. Activin A knockdown reduced both the expression of osteoblast-specific markers and matrix mineralization at day 21. In addition, we showed that in BM-MPCs under osteogenic induction, the activin A:follistatin ratio was significantly decreased at the end of the differentiation period compared with day 0. An activin-dominant microenvironment thus appears to be critical for the early stage of osteoblastic differentiation. Compared with BM-MPCs, M-MPCs, and DP-MPCs, T-MPCs with the lowest activin A level also exhibit the lowest chondrogenic potential. In addition, we observed that activin A knockdown in BM-MPCs significantly affects their chondrogenic potential. The activin A expression level in MPCs thus appears to govern both their chondrogenic and osteogenic potential. Our study is consistent with previous studies showing that during skeletal development, activin A is involved early at the beginning of the cascade of events promoting mesenchymal condensations and thus chondrogenic/osteogenic differentiation [54,55]. At the early phase of adipogenesis, activin A treatment suppresses differentiation, which could be rescued with follistatin [34]. This inhibitory effect of activin A on adipogenesis of progenitor cells acts through suppression of the adipocyte transcriptional factor cascade [33]. Our results show that activin A knockdown in BM-MPCs undergoing adipogenic induction results in a significant increase in their adipogenic activity. Taken together, these observations raise the possibility that the activin A expression level in MPCs is a key determinant in their commitment to a specific mesenchymal lineage, and activin signaling and activin target genes might be crucial for controlling MPC multipotency.

Another functional characteristic of MPCs or mesenchymal stem cells (MSCs) is their capacity to inhibit the proliferation of activated T cells [45]. The precise mechanisms underlying the immunosuppressive effect of MPCs/MSCs still remain to be clarified. The suppression of T-cell proliferation stimulated by allogeneic lymphocytes, dendritic cells, and mitogens, such as PHA or concanavalin A, has been well documented (for review, see [3]). Although controversial results have been reported, more convergent data now suggest that MPCs/MSCs may act by suppressing the differentiation of monocytes into mature dendritic cells (DC) thus impairing the stimulation of T cells [46,56,57]. Interestingly, in addition to its defined role in embryogenesis and erythroid cell differentiation in bone marrow, activin A has been shown to exert antiinflammatory effects [58,59]. Activin A was suggested to play a functional role in the suppression of inflammatory or immune processes [60]. More recently, activin A has been described as a novel DC-derived cytokine that potently attenuates CD40 ligand-specific cytokine and chemokine production. Blocking autocrine activin-A signaling in DCs by using its antagonist, follistatin, enhanced production of DC cytokines (IL-6, IL-10, IL-12p70, and TNF-α) [61]. These correlations between activin A and immunosuppression support our data showing that lower level of activin A produced by T-MPCs, compared with other MPC types, corresponds to their significantly lower immunosuppressive potential. However, M-MPCs and DP-MPCs, with an activin A:follistatin ratio similar to that of BM-MPCs, are also significantly less immunosuppressive than BM-MPCs. This might be explained by the fact that whereas DC maturation inhibition is certainly a mechanism by which MPCs display suppressive activity, it probably is not the main mechanism.

In addition, in this report, we show that (a) MPCs express the embryonic transcription factor Sox2 at a very low level and that its expression is enhanced on treatment with 10 ng/ml of activin A; (b) M-MPCs exhibit higher expression levels of both activin A and Sox2 compared with BM-MPCs, T-MPCs, and DP-MPCs; and (c) the downregulation of the activin A:follistatin ratio in differentiated BM-MPCs parallels a reduction in Sox2 expression level. Recent findings with hESCs show that activin A induces the expression of Oct4, Nanog, Nodal, Wnt3, bFGF, and FGF8, and is necessary and sufficient for the maintenance of self-renewal and pluripotency of hESCs [19]. In MSCs, Oct4 knockdown results in markedly decreased levels of Nanog and Sox2 expression [62]. Together, these studies strongly suggest that activin A is a key regulator in the maintenance of pluripotency of stem cells acting upstream of the master embryonic transcription factors Oct4, Nanog, and Sox2.

## Conclusions

MPCs are defined by their ability to give rise to mesodermal cell types. Multipotency in cultured MPCs derived from various tissues results from the integration of multiple signals to preserve this identity. In demonstrating a requirement of activin A, we have shown a novel candidate signaling mechanism for the maintenance of multipotency of MPCs.

## Abbreviations

AB: alcian blue; AGN: aggrecan; ALP: alkaline phosphatase; BM: bone marrow; BMP: bone morphogenetic protein; CFUs: colony forming units; COL2: collagen type II; DC: dendritic cell; DMEM: Dulbecco's modified Eagle's medium; DP: dental pulp; ESC: embryonic stem cell; FACS: fluorescence-activated cell sorting; FBS: fetal bovine serum; FGF: fibroblast growth factor; FLS: fibroblast-like synoviocyte; FS: follistatin; GAPDH: glyceraldehyde-3 phosphate dehydrogenase; HBSS: Hank's balanced salt solution; IBMX: 3-isobutyl-1-methylxanthine; LPL: lipoprotein lipase; M: muscle; MEM: minimum essential medium; MPC: mesenchymal progenitor cell; MSC: mesenchymal stem cell; OC: osteocalcin; PBMC: peripheral blood mononuclear cell; PBS: phosphate-buffered saline; PHA: phytohemagglutinin; PPAR-γ: peroxisome proliferator-activated receptor-γ; T: palatine tonsil; TGF-β: transforming growth factor-β.

## Competing interests

The authors declare that they have no competing interests.

## Authors' contributions

FD performed experimental work and analyzed and prepared the data and manuscript. WMJ procured the M-MPCs. BEB helped with the siRNA transfection. SJ procured the T-MPCs. YS performed the histology and the immunohistochemistry. GTJH procured the D-MPCs. RST participated in the experimental design and data analysis, prepared the manuscript, and supervised the project. All authors read and approved the final manuscript.
